# Supplementary material for: Manipulable preoperative factors affecting surgical outcomes of iStent inject W, particularly the type of antiglaucoma medications
Source: PLoS One. 2025 Dec 5;20(12):e0333882. doi: 10.1371/journal.pone.0333882 (PMC12680220; doi:10.1371/journal.pone.0333882)
Supplement: S1 Table — “Medication-free” was defined as GMS = 0 at 12 months. ARD was calculated as P(med-free | medication use) – P(med-free | no medication use). NNT (Number Needed to Treat) is only shown when ARD > 0. CI = confidence interval. (DOCX) [file pone.0333882.s001.docx]

Medication-free rates and ARD at 12 months according to preoperative medication use

| preoperative medication | n (med use) | Med-free % (med use) | n (no med use) | Med-free % (no med use) | ARD (95% CI) | NNT |
| --- | --- | --- | --- | --- | --- | --- |
| PG | 62 | 22.6% | 10 | 50.0% | -27.4% (-55.1 to 1.5) | - |
| BB | 42 | 21.4% | 30 | 33.3% | -11.9% (-32.3 to 8.3) | - |
| CAI | 33 | 9.1% | 39 | 41.0% | -31.9% (-48.6 to -11.8) | - |
| AA | 29 | 10.3% | 43 | 37.2% | -26.9% (-43.3 to -6.3) | - |
| ROCK inhibitor | 11 | 36.4% | 61 | 24.6% | +11.8% (-12.6 to 41.5) | 8.5 |
