# Supplementary material for: Manipulable preoperative factors affecting surgical outcomes of iStent inject W, particularly the type of antiglaucoma medications
Source: PLoS One. 2025 Dec 5;20(12):e0333882. doi: 10.1371/journal.pone.0333882 (PMC12680220; doi:10.1371/journal.pone.0333882)
Supplement: Attachment — Submitted filename: Response to Reviewers.docx [file pone.0333882.s003.docx]

**Response to reviewers**

The following pages present the verbatim comments of the reviewer and editor in black**;** our responses to each point are printed in red**.**  We highlighted *words* we modified or added in this version of our manuscript. We hope our changes satisfactorily address the comments.

Reviewer #1

1. Study design and ethical considerations;

It is stated that “Preoperative topical antiglaucoma medications were generally standardized according to the guidelines of relevant academic societies,” but given this, isn't the number of combinations of eye drops shown in Figure 1 excessive? An overview of the guidelines should be provided.

We sincerely appreciate the reviewer’s thoughtful question regarding the use of preoperative topical antiglaucoma medications and the combinations shown in Figure 1.

As stated in the manuscript, the administration of antiglaucoma medications in our study was generally based on the treatment algorithms recommended by major academic societies, including the World Glaucoma Association, the American Academy of Ophthalmology, and the Japan Glaucoma Society. All three recommend PG as the first-line therapy for glaucoma, based on their superior IOP-lowering efficacy and favorable safety profile. When monotherapy is insufficient, adjunctive therapy is recommended. [30, 31, 32]

In particular, the Japan Glaucoma Society guidelines recommend BB and EP2 receptor agonists as preferred second-line options following PG, due to their balance of efficacy and tolerability. Additional antiglaucoma medications such as CAI, AA, ROCK inhibitors, alpha1-blockers, ion channel openers, and parasympathomimetic agents are also considered appropriate as second- or later-line therapies depending on the patient's clinical condition. [32]

Consistent with these recommendations, Figure 1 shows that the vast majority of patients in our study were prescribed PG and/or BB as initial medications. When sufficient IOP reduction was not achieved, or when adverse effects occurred with these antiglaucoma medications, additional medications from other classes were added or substituted. This escalation strategy explains the wide variety of drug combinations observed in Figure 1 and reflects real-world clinical practice guided by current glaucoma treatment guidelines and individualized care.

We hope this explanation clarifies that the diversity of medication combinations observed in our study is aligned with current guideline-based treatment approaches and individualized patient care.

Action: We added a brief explanation in the Discussion section to clarify that the combinations of antiglaucoma medications used in this study were in accordance with major glaucoma treatment guidelines.

Revised manuscript, page 14, lines 4-13, in the Discussion section:

*According to the guidelines of the World Glaucoma Association, American Academy of Ophthalmology, and Japan Glaucoma Society, PG is recommended as the first-line therapy for glaucoma because of its superior IOP-lowering effects.[30-32] When monotherapy is insufficient, adjunctive treatment with BB or EP2 receptor agonists is typically recommended. This can be followed by additional agents if required, such as CAI, AA, or ROCK inhibitors, depending on the clinical context. [32] In our study, nearly all patients were prescribed PG or BB as the initial treatment. When sufficient IOP reduction was not achieved or when tolerance or adverse effects occurred with these medications, additional medications from other classes were added or substituted in a stepwise manner. This guideline-based approach explains the variety of antiglaucoma medication combinations shown in Fig. 1A and 1 B.*

2. LMM analysis of the relationship between preoperative factors and surgical outcomes;
Fixed effects and random effects are shown, but their values differ greatly. How should this result be interpreted? In other words, what does the large difference in percentages mean, and how should the results be interpreted?

We sincerely appreciate the reviewer’s insightful question. We agree that the difference between the variance explained by the fixed effects (Marginal R²) and by the combined fixed and random effects (Conditional R²) indicates that individual patient characteristics contribute to the variability in surgical outcomes (IOP and GMS) after ISIW implantation.

Thus, we believe that it is essential to provide treatment that accounts for such individual differences, and our results also suggest that the preoperative use of two types of antiglaucoma medications (BB or ROCK inhibitor) could reduce the number of antiglaucoma medications required after ISIW implantation.

**Action:** We revised the sentence to the Discussion section as follows:

Revised manuscript, page 14, lines 20-23, in the Discussion section:

*The difference between the variance explained by the fixed effects (Marginal R²) and the combined fixed and random effects (Conditional R²) in our LMM analysis suggests inter-individual variability in surgical outcomes. While it is essential to provide a treatment that accounts for such individual differences, our results also suggest* that the preoperative use of two types of antiglaucoma medications (BB or ROCK inhibitor) could reduce the number of antiglaucoma medications required after ISIW implantation.

We thank the reviewer for this important point, which helped us strengthen the interpretation of our findings.

3. Discussion;

It is stated that “Regarding preoperative antiglaucoma medications as one of the manipulatable factors by ophthalmologists, the LMM analysis for postoperative GMS showed a statistically significant positive coefficient for BB and ROCK inhibitor,” however, the intraocular pressure-lowering effects of each eye drop, the duration of use, and the order of administration are unclear. Based on these results, it is unclear how these eye drops should be used, and whether these results are clinically meaningful.

We sincerely appreciate the reviewer’s insightful comment regarding the clinical interpretation of the effects of each preoperative antiglaucoma medication, including their IOP-lowering efficacy, duration of use, and order of administration.

As the reviewer pointed out, these factors are highly relevant to interpreting the significance of our findings. Several previous meta-analyses have reported on the IOP-lowering efficacy of individual antiglaucoma medications. For example, a network meta-analysis by Li et al. (2015) estimated that after 3 months of monotherapy, PGs such as bimatoprost, latanoprost, tafluprost, and travoprost reduced IOP by approximately 4.3–5.6 mmHg; BBs such as timolol and carteolol reduced IOP by around 3.4–3.7 mmHg; CAIs such as brinzolamide and dorzolamide reduced IOP by around 2.4 mmHg; and AAs such as brimonidine and apraclonidine reduced IOP by around 2.5-3.5 mmHg. Another meta-analysis by Lee et al. (2022) reported that latanoprost (PG) showed a significantly greater IOP-lowering effect than netarsudil (ROCK inhibitor) after 4–6 weeks of monotherapy (mean difference 0.95 mmHg). Additionally, Li et al. (2017) demonstrated that PG monotherapy had the strongest IOP-lowering effect, followed by BB, AA, and CAI, and that dual therapies combining PG with other classes (e.g., PG + BB, PG + CAI, PG+AA) were more effective than combinations of non-PG drugs. However, none of these studies reported on the clinical effects based on the order of medication initiation.

We also acknowledge, as noted in our Discussion, that Okuda et al. (2022) reported that prolonged use of certain antiglaucoma medications may negatively affect surgical outcomes by inducing disuse atrophy of the conventional aqueous outflow pathway. Specifically, they suggested that long-term administration of drugs that either enhance uveoscleral outflow or suppress aqueous humor production—such as PGs, BBs, CAIs, and AAs—could reduce physiological flow through the conventional Schlemm’s canal pathway, potentially diminishing the surgical success of μTLO. This further supports the importance of evaluating the duration of preoperative medication use in future studies.

In our study, because it was retrospective and most patients were referred from local clinics to our hospital as a regional core center, we could not obtain accurate information on the onset and duration of preoperative antiglaucoma medication use, nor determine the extent to which IOP had already been lowered prior to surgery by these medications.

While our analysis identified statistically significant associations between preoperative BB or ROCK inhibitor use and postoperative GMS reduction, we acknowledge that the clinical significance of these findings remains limited due to the lack of data on medication duration, sequencing, and IOP response prior to surgery.

To further address the reviewer’s comment, we additionally tested models including preoperative GMS (PreGMS) as a fixed effect together with each preoperative medication. However, because PreGMS is the sum of all preoperative medications, it showed strong correlations with individual drug variables (e.g., correlation coefficient = 0.77 with CAI, 0.67 with BB, 0.57 with ROCK), which resulted in multicollinearity and led to unstable estimates, including the dropping of ROCK inhibitor from the model. We therefore concluded that including PreGMS simultaneously with individual drug variables was not appropriate and retained the models without PreGMS to avoid collinearity.

We agree with the reviewer that understanding the duration of preoperative antiglaucoma medication use and treatment sequence could improve the interpretability and clinical applicability of our findings. More precise evaluation of preoperative medication history could help inform optimal therapeutic timing and strategies prior to surgical intervention. Therefore, we have added a statement to the Discussion section to acknowledge this important limitation and to emphasize the need for future prospective studies that can account for medication duration and sequencing.

**Action:** We revised the sentence to the Discussion section, and we also divided the Limitations into separate paragraphs to improve readability.

Revised manuscript, page 17, lines 9-17, in the Discussion section:

*Since our study was retrospective, and most patients were referred from local clinics to our hospital due to it being a regional core center, we could not obtain accurate information on the onset and duration of preoperative antiglaucoma medication use or determine the extent to which IOP had already been lowered through the use of these medications prior to surgery. We also attempted an additional analysis, including the preoperative GMS (PreGMS) as a fixed effect, along with individual medication variables. However, because the PreGMS represents the sum of all preoperative medications, it was strongly correlated with each individual drug variable, leading to multicollinearity and unstable model estimates. Therefore, PreGMS was not included in the final model.*

Revised manuscript, page 18, lines 7-9, in the Discussion section:

*Thus, further prospective studies with detailed longitudinal tracking of the duration of preoperative medication use are necessary to better inform clinical decision making.*

4. Discussion;

Although it is stated that “when used in combination with PG, BB may have some indirect effects on the trabecular meshwork, leading to lower expression of pro-fibrotic genes in the trabecular meshwork compared to either drug alone.[33],” since iStent forms a bypass with the Schlemm's canal, changes in TM may not be very relevant. In other words, the discussion of PG + BB does not explain why BB was useful. There is insufficient discussion regarding the usefulness of BB.

We sincerely appreciate the reviewer’s valuable comment.

We have re-evaluated our dataset and found that the mean preoperative IOP was slightly higher in all antiglaucoma medication groups compared to the overall cohort. Specifically, the mean preoperative IOP was 15.46 mmHg in the PG group (n = 103), 15.94 mmHg in the BB group (n = 64), 16.08 mmHg in the CAI group (n = 51), 16.22 mmHg in the AA group (n = 45), and 16.37 mmHg in the ROCK inhibitor group (n = 19), whereas the overall mean IOP was 15.43 mmHg. These data indicate that higher preoperative IOP was not unique to the BB group. However, only BB and ROCK inhibitor use were statistically significant in the LMM analysis for GMS change.

This discrepancy suggests that preoperative IOP alone does not fully explain the association between medication use and surgical outcome. We hypothesize that the statistical significance observed for BB use may reflect the distinct pharmacological and clinical characteristics of BB. First, BBs are widely prescribed in combination with PG, and prior studies have suggested that this combination may suppress pro-fibrotic gene expression in the trabecular meshwork more effectively than either drug alone. Second, BBs act primarily by suppressing aqueous humor production without altering the structural integrity of the trabecular outflow pathway, which may enhance the effectiveness of ISIW implantation. Finally, we note that among the 57 eyes that received PG+BB preoperatively, BB was not reintroduced at 12 months postoperatively in 32 eyes, suggesting that the observed GMS improvement may partially reflect the IOP-lowering effect of ISIW, which in turn reduced the need for BB and other medications.

We also note the lack of direct clinical studies evaluating the relationship between BB use and MIGS outcomes, and we agree that further prospective research is needed to clarify whether BB use plays a causative or associative role in postoperative success.

**Action**: We have revised the Discussion section to clarify these points and acknowledge the speculative nature of the previous interpretation.

Revised manuscript, page 16, lines 7-15, in the Discussion section:

*We also reevaluated our dataset and found that the mean preoperative IOP was slightly higher in all antiglaucoma medication groups than in the overall cohort. Specifically, the mean preoperative IOP was 15.46 mmHg in the PG group (n = 103), 15.94 mmHg in the BB group (n = 64), 16.08 mmHg in the CAI group (n = 51), 16.22 mmHg in the AA group (n = 45), and 16.37 mmHg in the ROCK inhibitor group (n = 19), while the overall mean IOP was 15.43 mmHg. Although all groups showed slightly higher preoperative IOPs than the overall average, only BB and ROCK inhibitor use was statistically significant in the LMM analysis for GMS changes. This discrepancy suggests that preoperative IOP alone does not fully explain the surgical outcomes.*

Revised manuscript, page 17, line 22 to page 18, line 2, in the Discussion section:

*Although our study identified a statistically significant association between preoperative BB use and GMS improvement after ISIW implantation, there is currently a lack of direct clinical evidence to evaluate the relationship between BB use and surgical outcomes in MIGS. Accordingly, the observed significance of BB use in our LMM analysis should be interpreted with caution as it may reflect an associative rather than a causal effect.*

Reviewer #2

Major points:

1) In the introduction, authors include that “Topical antiglaucoma medications are the main methods to reduce IOP, and surgical treatments have generally been considered in eyes with uncontrolled glaucoma by topical antiglaucoma medications only.[2]”
Authors additionally include that preoperative IOP was an average of approximately 15mmHg.
Is this not considered well-controlled IOP? Why proceed with surgery?
Did a substantial number of these patients have NTG? If there was a substantial number of patients with NTG, was this glaucoma type related to any differences in outcomes?

We sincerely appreciate the reviewer’s important question regarding the surgical indication. As described in the Introduction, lowering IOP is the only evidence-based treatment for glaucoma, and previous reports have shown that ISIW implantation can achieve an additional IOP-lowering effect of approximately 1.8–2.7 mmHg at 12 months postoperatively and decrease in antiglaucoma medications.

In our study, ISIW implantation was performed for glaucoma patients undergoing cataract surgery in order to achieve greater IOP-lowering effects compared to cataract surgery alone. As our hospital is a university hospital, many patients were referred from other clinics and had already started topical antiglaucoma medications before the initial visit. Therefore, we did not have data on their untreated baseline IOP, and we were unable to precisely identify how many patients had normal-tension glaucoma.

If a substantial number of patients had normal-tension glaucoma, it is reasonable to assume that greater IOP reduction would be needed to prevent visual field progression compared to patients with high-tension glaucoma. This could mean that the observed decrease in postoperative antiglaucoma medications may be underestimated in our study.

In response to this comment, we have revised the Materials and Methods and the Results to clarify this point and to help readers better understand the background of our patient population.

**Action:** We have added the following clarifications to the manuscript:

Revised manuscript, page 6, lines 13-17, in the Patients selection and data acquisition section in the Materials and Methods:

*As our hospitals are university referral centers, many patients had already started topical antiglaucoma medications at the time of referral, and untreated baseline IOP data were not available. Therefore, the exact proportion of patients with NTG could not be determined. As a result,* the changes of IOP and GMS were set to be the main outcome to evaluate the effects of ISIW implantation.

Revised manuscript, page 9, line 10-13, in the Patient demographics section in the Results:

*Because many patients were referred from other clinics and had already started topical antiglaucoma medications, untreated baseline IOP data were not available. Consequently, the exact proportion of patients with normal-tension glaucoma could not be determined.*

We thank the reviewer for this valuable comment, which helped us improve the transparency and interpretability of our study.

2) The meaning of this statement is unclear:

“The sample size was insufficient, as it included only patients who did not voluntarily request MIGS.” Insufficient to determine what and insufficient in what way? Authors make conclusions at the end of the study. Are these conclusions supported by the sample size?

We sincerely appreciate the reviewer’s thoughtful comment.

We acknowledge that the original sentence was unclear. We used the phrase “The sample size was insufficient” to explain that it was not feasible to include a placebo group in this study, since introducing a non-MIGS (placebo) group would have required an appropriately larger sample size. We understand the importance of including a placebo group for accurate comparisons. However, the benefit of MIGS in lowering IOP has already been well established, and intentionally creating a placebo group for patients with glaucoma would not have been ethically acceptable.

Therefore, the sample size was insufficient to allow for randomization or a direct comparison with a placebo group. Moreover, considering the number of fixed effects included in the LMM analysis, we believe that setting up an additional placebo group would have required a similar number of eyes as in this study to maintain sufficient statistical power.

Nonetheless, we agree that larger, multicenter, prospective studies with appropriate controls would be valuable to further validate and expand upon these findings in the future.

**Action:** We revised the sentence in the Patients selection and data acquisition section in the Materials and Methods as follows:

Revised manuscript, page 5, line 23, to page 6, line 2, in the Patients selection and data acquisition section in the Materials and Methods

The inclusion of a placebo group was not feasible in this study because the benefit of MIGS in reducing IOP has already been well established*, and intentionally creating a placebo group for patients with glaucoma would not have been ethically acceptable. Moreover, introducing a placebo group would have required an appropriately larger sample size, especially considering the number of fixed effects included in the LMM analysis; however, the number of eligible patients who did not wish to undergo MIGS was too small to allow for meaningful comparison or randomization.*

We thank the reviewer for pointing this out and helping us improve the clarity and transparency of our manuscript.

3) Authors include in the Discussion,

“ROCK inhibitors have pharmacological effects such as relieving outflow resistance in the trabecular meshwork, which enhances aqueous humor drainage through the pathway,[30, 31] and inhibiting fibroblast proliferation, which helps reduce scarring post-surgery.[32] These effects might have contributed to improved surgical outcomes after ISIW implantation. No clinical reports have directly indicated a relationship between the use of BB and the effects of surgical treatments associated with conventional outflow pathways, including laser therapy. Interestingly, when used in combination with PG, BB may have some indirect effects on the trabecular meshwork, leading to lower expression of pro-fibrotic genes in the trabecular meshwork compared to either drug alone.[33]”

This text seems to suggest that ROCK inhibitors and beta blockers are likely to be helpful as preoperative medications to decrease IOP and/or GMS. However, it seems that they had no significant effect on postoperative IOP and caused significant increases (not improvement) in postoperative GMS.

Additionally, in the abstract, authors include,

“Preoperative factors, particularly medication use, influenced outcomes, indicating that β blockers or Rho kinase inhibitors may reduce the need for postoperative antiglaucoma medications.”

Based on the data shown, it seems that β blockers or Rho kinase inhibitors may increase (not reduce) the need for postoperative antiglaucoma medications. Is this correct? If so, please adjust the manuscript to clarify and justify the increase rather than decrease of GMS as a result of preoperative ROCK inhibitors.

We sincerely appreciate the reviewer’s important comment, which helped us clarify the interpretation of our results for GMS change.

As shown in Figure 2B, we compared preoperative and postoperative GMS using Tukey’s HSD test and found that GMS significantly decreased at all observation points up to 12 months postoperatively (p < 0.05). Similarly, a significant decrease in IOP was observed in Figure2A from 3 to 12 months postoperatively (p<0.05).

In Table 3, we presented the results of the LMM analysis for GMS change. Here, the objective variable was the change in GMS calculated as the difference between preoperative and postoperative GMS (preoperative minus postoperative). Therefore, a positive coefficient for a fixed effect indicates an increase in the change value, which means that the postoperative GMS tends to be lower (a larger decrease). The same interpretation applies to IOP change shown in Table 2.

Thanks to the reviewer’s insightful comment, we realized that the relationship between Figure 2, Tables 2 and 3, and the interpretation of the coefficients could be difficult for readers to follow.

To address this, we have revised the Data analysis section in the Materials and Methods and the Discussion section to clarify the calculation and interpretation of the change values in the LMM analysis.

**Action:** We revised the sentence in the Materials and Methods and the Discussion section as follows:

Revised manuscript, page 8, lines 3-6, in the Data analysis section in the Materials and Methods:

*Changes in IOP and GMS were defined as the preoperative value minus the postoperative value at each time point. Therefore, a positive coefficient indicated that the variable was associated with a greater reduction in postoperative IOP or GMS.*

Revised manuscript, page 15, lines 1-5 in the Discussion section:

LMM analysis *was performed using the change in GMS and IOP as objective variables, calculated as the difference between the preoperative and postoperative value (preoperative minus postoperative) at 3 to 12 months after surgery. This analysis* showed a negative coefficient value over postoperative days, although the value was apparently small compared with other variables.

We sincerely thank the reviewer for this valuable comment, which helped us improve the clarity and consistency of our manuscript.

4) Because PLOS one has a broad readership, can authors discuss how clinically meaningful the IOP/GMS changes these preoperative factors may allow is?

For example, how much might the average IOP change of ~0.5mmHg associated with preoperative IOP tend to affect outcomes clinically? How much might a GMS change of ~0.7 (associated with beta blockers) affect outcomes clinically?

This can help contextualize the magnitude of findings.

We sincerely appreciate the reviewer’s insightful comment regarding the clinical meaningfulness of the observed IOP and GMS changes.

With respect to IOP, even relatively small reductions may be clinically relevant. The Early Manifest Glaucoma Trial (EMGT) reported that each 1 mmHg reduction in IOP from baseline was associated with an approximately 10% decrease in the risk of glaucoma progression. [2004, Leske]

Regarding GMS, we conducted an additional exploratory analysis to contextualize the clinical significance of medication reduction. We defined “medication-free” as GMS = 0 at 12 months and calculated the absolute risk difference (ARD) in medication-free rates between patients with and without each class of preoperative antiglaucoma medication. For example, in eyes preoperatively treated with beta-blockers, the medication-free rate at 12 months was 21.4% compared with 33.3% in those without beta-blocker use (ARD = –11.9%, 95% CI: –32.3% to 8.3%). For ROCK inhibitors, the medication-free rate was 36.4% versus 24.6% (ARD = +11.8%, 95% CI: –12.6% to 41.5%). These results suggest that beta-blocker use may be associated with a lower likelihood of being medication-free after surgery, whereas ROCK inhibitor use may be associated with a higher likelihood, although the wide confidence intervals indicate substantial uncertainty.

In summary, we believe that while the observed differences in IOP and GMS may appear numerically small, they may still carry potential clinical implications, particularly in the long-term management of glaucoma. However, as our exploratory ARD analysis focusing on GMS change shows, the confidence intervals are wide and the results should be interpreted with caution.

Medication-free was defined as GMS = 0 at 12 months postoperatively. ARD was calculated as P(med-free | medication use) – P(med-free | no medication use). NNT (Number Needed to Treat) is only shown when ARD > 0. CI = confidence interval; ARD = absolute risk difference; NNT = number needed to treat.

**Action:** We revised the sentence in the Results and the Discussion section as follows:

Revised manuscript, page 13, line 16, to page 14, line 1, in the Results section:

*To further contextualize the clinical meaningfulness of postoperative medication reduction, we performed an exploratory analysis. “Medication-free” was defined as GMS = 0 at 12 months, and the absolute risk difference (ARD) in medication-free rates was calculated as P(med-free | medication use) – P(med-free | no medication use). NNT (Number Needed to Treat) is only shown when ARD > 0. As shown in Supplementary Table 1, the medication-free rate at 12 months was lower in patients preoperatively treated with beta-blockers (21.4% vs. 33.3%, ARD = –11.9%, 95% CI: –32.3% to 8.3%), and higher in those treated with ROCK inhibitors (36.4% vs. 24.6%, ARD = +11.8%, 95% CI: –12.6% to 41.5%).*

***Supplementary Table 1: Medication-free rates and ARD at 12 months according to preoperative medication use***

Revised manuscript, page 15, lines 9-17 in the Discussion section:

The LMM analysis also showed a positive coefficient for preoperative IOP. *Thus*, our surgical outcomes of ISIW implantation resulted in better improvement of postoperative IOP in eyes with higher preoperative IOP, which was consistent with previous reports on iStent.23-25 *Furthermore, the Early Manifest Glaucoma Trial demonstrated that each 1 mmHg reduction in IOP from baseline was associated with an approximately 10% decrease in the risk of glaucoma progression.[33] This indicates that even relatively small IOP reductions may be clinically relevant. Nevertheless,* our study design does not allow for a strong discussion of IOP reduction because it allows for changes in the type of antiglaucoma medications based on the visual field and IOP.

Revised manuscript, page 18, lines 2-7, in the Discussion section:

*Our exploratory ARD analysis of the GMS indicated that beta-blocker use was associated with a lower likelihood of achieving medication-free status, whereas ROCK inhibitor use was associated with a higher likelihood, although the wide confidence intervals highlight the uncertainty of these findings. These results suggest that, although the absolute differences may appear numerically small, they may still have potential clinical implications for long-term glaucoma management.*

5) Baseline IOP in Table 1 is different from preoperative IOP in the abstract and results (page 10, first sentence.)

Why is this? Is “baseline” and “preoperative” the same category or different categories?

We sincerely appreciate the reviewer’s insightful comment.

The reason for this discrepancy is that Table 1 presents baseline IOP for all included cases that met the inclusion criteria (114 eyes of 114 patients). In contrast, the IOP and GMS values reported in the Abstract, as well as in the Pre- and post-operative changes of IOP and GMS section of the Results, are based specifically on the subset of 72 eyes of 72 patients with complete 12-month follow-up data.

We recognize that this difference may cause confusion for readers. To address this, we have added a clarifying note to the sentence in the Abstract to specify that both the IOP and GMS data in this part are based on the 72 cases with 12-month follow-up. Additionally, the Results section already states that these analyses were performed using data from 72 eyes of 72 patients, so no further clarification was needed there.

**Action:** We have separated this part into a new paragraph in the Abstract section to clarify the difference between the overall baseline data and the data used for the 12-month follow-up analysis, and we have added the following note to the Abstract section:

Revised manuscript, page 2, lines 14-15, in the Abstract section:

*The IOP and GMS values were based on a subset of 72 eyes with 12-months of complete follow-up data.*

We thank the reviewer for pointing this out and helping us improve the clarity and transparency of our manuscript.

Minor points:

1) Could authors provide comment on whether or not preoperative medications are likely to affect surgical outcomes after MIGS other than ISIW? Is there anything in particular about the mechanism of ISIW that is likely to be influenced differently by these preoperative medications compared to other iStents or other MIGS?

We sincerely appreciate the reviewer’s thoughtful question regarding the potential influence of preoperative antiglaucoma medications on surgical outcomes following MIGS other than ISIW, and whether there is anything specific about the mechanism of ISIW that might result in a differential effect.

As noted in our Discussion, our results suggest that the use of certain preoperative antiglaucoma medications, particularly ROCK inhibitors, may influence the surgical outcomes of ISIW implantation. This is likely due to their pharmacological effects on the trabecular outflow pathway, including relieving outflow resistance in the trabecular meshwork and reducing postoperative scarring by inhibiting fibroblast proliferation. [2001, Honjo; 2001, Rao; 2016, Futakuchi]

There is emerging evidence that similar effects may be observed with other trabecular-targeting MIGS. For example, a retrospective study by Okuda et al. (2023) reported that eyes treated with ripasudil prior to microhook ab interno trabeculotomy (µTLO) achieved significantly higher surgical success rates than nonusers. Notably, the author suggested that, unlike most other antiglaucoma medications (e.g., PG, BB, CAI, and AA), ROCK inhibitors may not induce disuse atrophy of the conventional trabecular outflow system and may even help preserve or improve its function. [2023, Okuda]

Furthermore, a study reported that the probability of achieving surgical success after trabeculotomy was 100% in ripasudil-effective eyes, highlighting the potential clinical value of this medication in optimizing outcomes for procedures targeting the conventional outflow pathway. [2019, Goda]

Based on these findings, we believe that the key factor is the shared target site of action between ISIW and ROCK inhibitors, rather than a mechanism specific to ISIW.

**Action:** We revised the sentence in the Discussion section as follows:

Revised manuscript, page 16, lines 5-7, in the Discussion section:

These effects might have contributed to improved surgical outcomes after ISIW implantation, *which targets the trabecular outflow pathway, similar to ROCK inhibitors.*

In response to the reviewer’s comment, we reviewed the relevant section of the Discussion and found that the attribution of references was inaccurate. Specifically, reference [20], which reports the effectiveness of trabeculotomy in ROCK inhibitor–effective eyes, was mistakenly grouped with reference [21], which addresses selective laser trabeculoplasty. To correct this, we revised the sentence to clearly distinguish the type of surgery each study evaluated and to accurately assign the supporting references.

**Action:** We revised the sentence in the Discussion section as follows:

Revised manuscript, page 15, line 22, to page 16, line 2, in the Discussion section:

*Notably, one study reported higher effectiveness of trabeculotomy in ROCK inhibitor–effective eyes compared to other eyes in the study[20], and another study showed greater success of selective laser trabeculoplasty in ROCK inhibitor–effective eyes compared to other eyes in the study[21]. Furthermore, a separate* study reported a lower necessity of additional glaucoma surgeries after microhook ab interno trabeculotomy in the ROCK inhibitor-used group [22].

2) Tables 2 and 3 have the same titles. Is Table 3 intended to signify changes in GMS?

We sincerely appreciate the reviewer’s careful reading and valuable comment. As the reviewer correctly pointed out, the titles of Tables 2 and 3 were mistakenly the same in the original manuscript.

**Action:** We have corrected the title of Table 3 in the Results section as follows:

*“Results of the LMM Analysis for GMS Change.”*

We thank the reviewer for helping us improve the clarity and accuracy of our manuscript.

3) The following sentence is a fragment. Please correct this incomplete sentence or combine with the words in the next sentence.
"To improve surgical effects or reduce complications, variable glaucoma surgeries, including micro invasive glaucoma surgeries (MIGS) such as Trabectome,[5] Hydrus microstent,[6-8] Kahook Dual Blade.[9]
"suture, trabeculotomy[11] and iStent,[12] have been developed."

Thank you for your comments and careful review. We appreciate the reviewer's helpful comment. We understand the reviewer’s point that part of the text was fragmented.

**Action:** We revised the sentence in the Introduction section as follows:

Revised manuscript, page 3, lines 3-7 in the Introduction section:

To improve surgical effects or reduce complications, variable glaucoma surgeries, including micro invasive glaucoma surgeries (MIGS) such as Trabectome, [5] Hydrus microstent, [6-8] Kahook Dual Blade*, [9] gonioscopy-assisted transluminal trabeculotomy, [10] 360 degrees* suture trabeculotomy[11] and iStent, [12] have been developed.

4) The meaning of this statement is unclear:
“When both eyes of the same patient fulfilled these criteria, we used data from an earlier surgical date.”
Data from which eye was used when both eyes of the same patient fulfilled the criteria?

We sincerely appreciate the reviewer’s comment pointing out the ambiguity of this statement. We intended to convey that when both eyes of the same patient met the inclusion criteria, only one eye was included in the analysis to avoid duplication, and we selected the eye that underwent surgery first.

**Action**: To address this, we have clarified the patient selection criteria section and the Data analysis section of the Materials and Methods as follows:

Revised manuscript, page 5, lines 20-21, in the Patient selection and data section of the Materials and Methods:

When both eyes of the same patient fulfilled these criteria, *only the eye with the earlier surgical date was included, in order to avoid duplicate data from the same patient.*

Revised manuscript, page 7, line 24, to page 8, line1, in the Data analysis section of the Materials and Methods:

*Additionally, to minimize intraindividual bias, only one eye per patient was included in the final analysis when both eyes met the inclusion criteria.*

In addition, since only one eye per patient was included in the present study, we have revised the Data Analysis section accordingly to reflect this methodology.

Revised manuscript, page 8, lines 1-2, in the Data analysis section of the Materials and Methods:

*LMM can account for the fact that eyes may have different IOP and GMS values over time within the same individual.*

Upon re-examining the dataset in response to this comment, we found that in one out of 114 cases, the eye with the later surgical date was mistakenly included. We have corrected this and reanalyzed the data accordingly. As a result, some numerical values in the results changed slightly, and we have updated the relevant parts of the manuscript, including the main text, Tables 1, 2, and 3, and Figures 2A and 2B.

**Action**: We have updated the relevant numerical results, tables and figures as follows:

Revised manuscript, page 9, lines 3-6, in the Patient demographics section of the Results:

*At 6, 9, and 12 months after surgery, IOP data were available for 97 eyes of 97 patients (85%), 84 eyes of 84 patients (74%), and 75 eyes of 75 patients (66%), respectively. GMS data were available for 98 eyes of 98 patients (86%), 85 eyes of 85 patients (75%), and 77 eyes of 77 patients (68%), respectively.*

Revised manuscript, page 9, lines 14-15, in the Patient demographics section of the Results:

There were *66 (57.9%)* right eyes and *48 (42.1%)* left eyes. Demographic data are summarized in Table 1.

Revised manuscript, page 10, line 1, in Table 1:

Revised manuscript, page 11, lines 8-16, in the Pre- and post-operative changes of IOP and GMS section of the Result:

The preoperative IOP and GMS was 15.06 ± 3.51 mmHg and 2.46 ± 1.33, respectively. The preoperative MD (mean ± SD) was *-6.79 ± 3.94* dB. The postoperative IOP was *14.40 ± 5.38* mmHg the next day, *16.53 ± 4.74* mmHg 7 days later, and 14.12 ± 3.35 mmHg 1 month later. Compared to preoperative values, postoperative IOP decreased significantly to 12.22 ± 2.23 mmHg 3 months later, *12.92 ± 2.73* mmHg 6 months later, *12.81 ± 2.64* mmHg 9 months later, and 12.99 ± 2.45 mmHg 12 months later (p < 0.05) (Fig 2A). Postoperative GMS decreased significantly compared to preoperative values to 0.00 ± 0.00 the next day, 0.74 ± 1.29 7 days later, 1.15 ± 1.11 1 month later, 1.32 ± 1.31 3 months later, *1.39 ± 1.34* 6 months later, 1.40 ± 1.38 9 months later, and 1.60 ± 1.41 12 months later (P < 0.05) (Fig 2B).

Revised manuscript, page 12, lines 10-16, in the LMM analysis of the relationship between preoperative factors and surgical outcomes section of the Results:

The value of coefficient was *-0.00284 ± 0.000557* in postoperative days values (p < 0.01), *-0.0150 ± 0.0365* in preoperative MD values (*p = 0.682*), *0.533 ± 0.0496* in preoperative IOP values (p < 0.01), *-0.262 ± 0.639* in PG values (*p = 0.683*), *0.136 ± 0.369* in BB values (*p = 0.713*), *-0.0401 ± 0.398* in CAI values (*p = 0.920*), *-0.162 ± 0.380* in AA values (*p = 0.670*), and *0.136 ± 0.481* in ROCK inhibitor values (*p = 0.778*).

Revised manuscript, page 12, line 25, to page 13, line 4, in the LMM analysis of the relationship between preoperative factors and surgical outcomes section of the Results:

The value of the coefficient was *-0.000817 ± 0.000186* in postoperative days values (p < 0.01), -0.0293 ± 0.0235 in preoperative MD values (p = 0.215), *-0.0452 ± 0.0322* in preoperative IOP values (*p = 0.163*), *0.310 ± 0.421* in PG values (*p = 0.464*), *0.719 ± 0.240* in BB values (p < 0.01), *0.0434 ± 0.259* in CAI values (*p = 0.0976*), *0.292 ± 0.247* in AA values (*p = 0.240*), and 0.734 ± 0.313 in ROCK inhibitor values (p < 0.05).

Revised manuscript, page 12, line 18, in Table 2:

Revised manuscript, page 13, line 9, in Table 3:

Revised Figure 2A:

Revised Figure 2B:

This revision did not substantially alter the overall conclusions.

We thank the reviewer for helping us improve the clarity and accuracy of our study.

5) Please specify the Python package(s) used for LMM analysis.

We appreciate the reviewer’s helpful comment. We did not use any Python packages for the LMM analysis. Instead, we performed the LMM analysis using R software with the following packages:

lme4: for fitting linear mixed-effects models using the lmer function

lmerTest: for calculating p-values for fixed effects

MuMIn: for calculating R-squared values

emmeans: for obtaining estimated marginal means and confidence intervals

**Action:** We revised the sentence in Data analysis section of the Materials and Methods as follows:

Revised manuscript, page 7, lines 23-24, in the Data analysis section of the Materials and Methods:

To address potential bias from the inclusion of multiple time points for the same patient, we employed LMM analysis *using the R packages lme4, lmerTest, MuMIn, and emmeans* to assess changes in GMS and IOP.

6) Please use consistent terms to refer to beta blockers throughout the text (BB vs. β blocker) and ROCK inhibitors / Rho kinase inhibitors.

We appreciate the reviewer’s helpful comment regarding the consistency of terminology. In response, we have standardized the terms for antiglaucoma medications throughout the manuscript as follows:

Prostaglandin analog: PG

β blocker: BB

Carbonic anhydrase inhibitor: CAI

Alpha2-agonist: AA

Rho kinase inhibitor: ROCK inhibitor

We have revised the main text, the Figure 1 legend, and the Figure 1A and 1B accordingly. For Figure 1A, we used the abbreviation ROCK instead of ROCK inhibitor within the figure itself to improve readability, while the figure legend retains the full term for clarity.

**Action:** We have revised the relevant sections as follows:

Revised manuscript, page 2, lines 16-20, in the Abstract section:

LMM analysis showed significant coefficients for IOP change in relation to postoperative days and preoperative IOP, and for GMS change in relation to postoperative days, *β blockers (BB)*, and *Rho kinase inhibitors* *(ROCK inhibitors)*. Preoperative factors, particularly medication use, influenced outcomes, indicating that *BB* or *ROCK inhibitors were associated with a greater reduction in* the need for postoperative antiglaucoma medications.

Revised manuscript, page 3, lines 18-21, in the Introduction section:

The National Institute for Health and Care Excellence and American Academy of Ophthalmology recommend the *prostaglandin analog (PG)* as the first choice for glaucoma therapy due to its superiority in terms of IOP-lowering effects compared to other antiglaucoma medications.[2]

Revised manuscript, page 4, lines 1-2 in the Introduction section:

Preoperative use of *ROCK inhibitors* leads to better surgical outcome in eyes that underwent glaucoma surgeries.[20-22]

Revised manuscript, page 10, lines 3-5 in the Preoperative antiglaucoma medications section of the Result:

For preoperative glaucoma therapy, 27 (23.7%) eyes were medicated by *PG* only; 15 (13.2%) eyes by *~~the~~* PG, *BB*, carbonic anhydrase inhibitor(CAI), and AA; 13 (11.4%) eyes by PG and BB; and 10 eyes (8.8%) medicated PG, BB, CAI, AA, and ROCK inhibitor (Fig 1A).

Revised manuscript, page 11, line 2-5 in Figure 1 legend of the Result:

*PG* is most frequently used in 103 eyes (90.4%) including 27 eyes as single patterns and 76 eyes as combination patterns. For other types of antiglaucoma medications, *BB* is used in 64 eyes (56.1%), *CAI* in 51 eyes (44.7%), *AA* in 45 eyes (39.5%), and *ROCK inhibitor* in 19 eyes (16.7%). *~~PG, Prostaglandin analog; β, β blocker; CAI, Carbonic anhydrase inhibitor; α, Alpha2-agonist; ROCK, Rho kinase inhibitor.~~*

We sincerely thank the reviewers and editor(s) for their constructive comments, which have greatly improved the quality of our manuscript.
